# Prevention of catheter-related bladder discomfort – pudendal nerve block with ropivacaine versus intravenous tramadol: study protocol for a randomized controlled trial

**DOI:** 10.1186/s13063-016-1575-y

**Published:** 2016-09-13

**Authors:** Jing-yi Li, Ren Liao

**Affiliations:** 1 Department of Dermatovenereology, West China Hospital, Sichuan University, Chengdu, 610041 People’s Republic of China; 2 Department of Anesthesiology, West China Hospital of Sichuan University, 37 Guoxue Lane, Chengdu, 610041 Sichuan Province People’s Republic of China

**Keywords:** Catheter-related bladder discomfort, Pudendal nerve block, Tramadol, Randomized controlled trial

## Abstract

**Background:**

Catheter-related bladder discomfort (CRBD) is a common distressing symptom complex during the postoperative period, especially after urologic procedures with a relatively greater size urinary catheter. In this study, we will enroll male patients undergoing elective prostate surgery with urinary catheterization under general anesthesia, and we will compare the efficacy of pudendal nerve block (PNB) and intravenous tramadol in CRBD prevention.

**Methods/design:**

This trial is a prospective, randomized controlled trial that will test the superiority of bilateral PNB with 0.33 % ropivacaine compared with intravenous tramadol 1.5 mg/kg for CRBD prevention. A total of 94 male patients undergoing elective prostate surgery with urinary catheterization after anesthesia induction will be randomized to receive either bilateral PNB with 0.33 % ropivacaine (the PNB group) or intravenous tramadol 1.5 mg/kg (the tramadol group) after the completion of surgery. The primary outcome is the incidence of CRBD. The most important secondary outcome is the severity of postoperative CRBD, and other secondary outcomes include Numeric Rating Scale (NRS) score for postoperative pain; incidence of postoperative side effects such as postoperative nausea/vomiting, sedation, dizziness, and dry mouth; postoperative requirement for tramadol as a rescue treatment for CRBD and sufentanil as a rescue analgesic for postoperative pain; and NRS score for acceptance of an indwelling urinary catheter.

**Discussion:**

This trial is planned to test the superiority of PNB with 0.33 % ropivacaine compared with intravenous tramadol 1.5 mg/kg. It may provide a basis for a new clinical practice for the prevention of CRBD.

**Trial registration:**

ClinicalTrials.gov identifier NCT02683070. Registered on 11 February 2016.

**Electronic supplementary material:**

The online version of this article (doi:10.1186/s13063-016-1575-y) contains supplementary material, which is available to authorized users.

## Background

Catheter-related bladder discomfort (CRBD) in patients with urinary catheterization during operation is frequently reported in the postanesthesia care unit (PACU) [1]. It is a distressing symptom complex characterized as a burning sensation or stabbing pain with an urge to void or as discomfort from the suprapubic area to the urethra [2]. The condition of CRBD may aggravate postoperative pain, increase the incidence of postoperative complications, and result in prolongation of hospital stay [2–4].

The mechanism of CRBD is mediated by type 3 muscarinic receptor activation, which increases acetylcholine release and then causes the detrusor muscles of the bladder to contract involuntarily [5]. Antimuscarinic agents, including tramadol, tolterodine, oxybutynin, pregabalin, and ketamine, have been applied to treat CRBD in clinical practice [6–10]. However, in spite of their beneficial effect on CRBD, systemic administration of these agents is associated with side effects such as nausea or vomiting, sedation, dizziness, or other unpleasant complications, which may result in reduced quality of recovery. It is important in clinical practice to establish an effective treatment for CRBD with reduced adverse effects.

Anatomically, the afferent nerves of the urethra and bladder triangle are derived from sacral somatic nerves (S2–S4 segment) [5] and theoretically, prevention of CRBD should be achieved by blocking these nerves. The pudendal nerve, the main nerve of the perineum, is derived from the ventral rami of the second, third, and fourth sacral spinal nerves; innervates the urethral sphincter and muscles of the perineum and pelvic floor; and provides sensation to the penis in males and the clitoris in females, as well as to the urethra and bladder triangle [11, 12]. Clinically, pudendal nerve block (PNB) has been applied for analgesia of labor and/or vaginal birth [13], vaginal repair [14], sphincterotomy [15], and treatment of pudendal neuralgia [16], and the safety and effectiveness of PNB have been identified [12–16]. On the basis of the anatomical and physiological proof for nerve innervation, with the support of clinical experience, we designed this trial with the hypothesis that PNB has a CRBD-reductive effect with less side effects related to systemic administration of antimuscarinic agents than PNB with ropivacaine and intravenous tramadol, and we aim to provide a novel and effective way to reduce the incidence of CRBD and with a better outcome.

## Methods/design

### Design

**Fig. 1:**
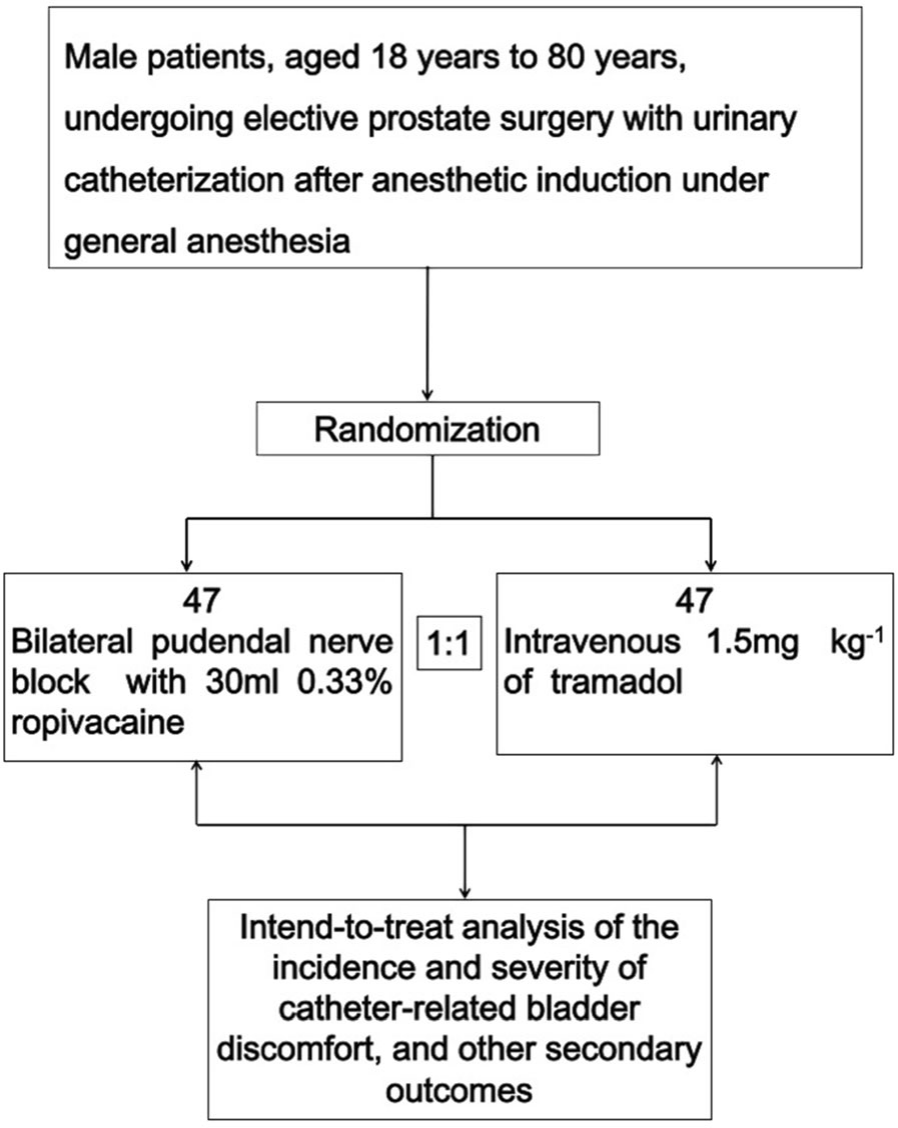
The pudendal nerve block trial algorithm

The PNB trial (ClinicalTrials.gov identifier NCT02683070) is an investigator-initiated, prospective, randomized controlled trial that will test the superiority of PNB with 0.33 % ropivacaine to intravenous tramadol 1.5 mg/kg for reduction of CRBD. It will be conducted in accordance with the principles outlined in the Declaration of Helsinki and will follow the Consolidated Standards of Reporting Trials (CONSORT) statement (http://www.consort-statement.org/). A brief flow diagram of the PNB trial is provided in Fig. 1, and a populated Standard Protocol Items: Recommendations for Interventional Trials (SPIRIT) checklist is provided in Additional file 1.

The study has no external financial sponsor, and there are no conflicts of interest in the study design, data collection, analysis, or results interpretation. Before we start the study, all of the participants, including physicians, interns, and nurses, will be educated and trained, and some of them will be responsible for follow-up. After the completion of this trial, the role of the participants, including trial designer, trial executor, and staff responsible for follow-up, will be stated in detail.

### Sample size calculation

The incidence of CRBD has been reported to range from 47 % to 90 % according to different types of surgery, and male sex has been identified to be an independent predictor in previous studies [1, 3]. We investigated 367 male patients in the PACU of the Department of Anesthesiology, West China Hospital, from 21 June 21 to 17 July 2015 and found that 56.1 % (206 of 367) of them complained of CRBD. On the basis of this information, a difference of 25 % (e.g., 30 % vs. 55 %) in the incidence of CRBD values between two treatment groups is considered to be clinically important. The sample size was calculated to compare two proportions with two-sample noninferiority or superiority; assuming the difference between two groups at a 5 % significance level and a power of 0.80, 37 patients in each group are required for a comparison within the groups. Considering an estimated 20 % dropout rate, 47 patients in each group, and 94 patients in total, are required in this study.

### Recruitment

A total of 94 patients undergoing elective prostate surgery in lithotomy position with urinary catheterization (16-French Foley catheter) while under general anesthesia will be enrolled at West China Hospital of Sichuan University.

### Randomization and blinding

A biostatistician in the Department of Anesthesiology, West China Hospital of Sichuan University, will perform randomization using SAS version 9.1 software (SAS Institute, Cary, NC, USA). For the purpose of blinding, the results of randomization and group allocation will be concealed in a nontransparent envelope and kept by a research assistant. After confirmation of the patient’s enrollment, the investigator will call the research assistant to get the allocated group and intervention by opening the envelope. The investigators, research assistant, and responsible physicians will not be blinded to the treatment assignment, and the patients, staff responsible for follow-up, and statisticians will be blinded to it.

### Study organization

The study will be supervised for data completeness and accuracy by the Department of Anesthesiology, West China Hospital, and RL will be responsible for data monitoring and alerting for serious complications. The data safety and monitoring board will be involved for the duration of the trial. There are no stop rules in this pilot study, and no preliminary analysis will be performed before the completion of this study.

### Enrollment criteria

The following are the inclusion criteria:

- Male patients aged 18–80 years
- Undergoing elective prostate surgery in lithotomy position with urinary catheterization while under general anesthesia

The exclusion criteria are as follows:

- History of bladder dysfunction, including overactive bladder, which is defined as urinary frequency more than three times in the night or more than eight times in 24 h [5]; neurogenic bladder; and bladder outflow obstruction
- Coagulation disorders
- History of vestibular dysfunction
- History of substance abuse
- Known allergy to ropivacaine, tramadol, or any other anesthetic agent
- History of postoperative delirium
- Impairment of cognitive function

### Interventions

In the PNB group, bilateral PNB with 30 ml of 0.33 % ropivacaine (15 ml for each side) will be performed after the operation is finished. In the tramadol group, intravenous tramadol 1.5 mg/kg will be administered after the operation is finished.

### Outcome measures

All patients will be extubated after completion of the operation and then transferred to the PACU for outcome evaluation. Patients will be asked directly and observed by staff responsible for follow-up who were blinded to the group assignments for outcomes at 0, 0.5, 1, 2, 4, and 6 h after patient arrival in the PACU and after extraction of the urinary catheter. A questionnaire designed to collect the outcome data is shown in Table 1.

**Table 1:**
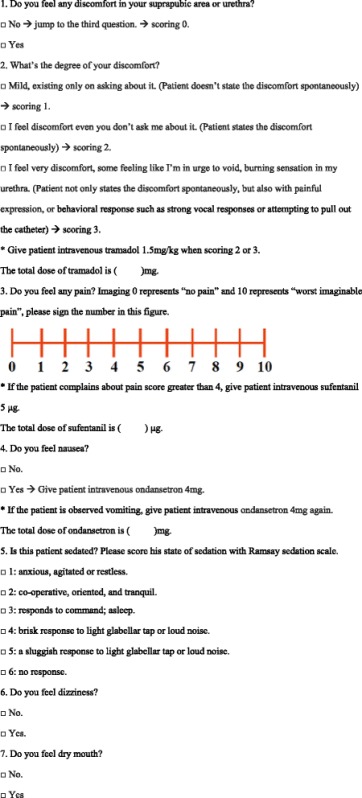
Questionnaire for pudendal nerve block trial outcome evaluation

### Primary outcome

The primary outcome is the incidence of postoperative CRBD.

### Secondary outcomes

The following are the secondary outcomes of interest in the trial:

1. The most important secondary outcome is the severity of postoperative CRBD, which will be assessed with a 4-point scale as follows: 0 = no CRBD; 1 = mild CRBD, existing only upon asking about it; 2 = moderate CRBD, stated by the patient spontaneously; and 3 = severe CRBD, demonstrated by behavioral response such as strong vocal responses or attempting to pull out the catheter [2]. When the severity of CRBD is evaluated as 2 or 3, the patient will be administered intravenous tramadol 1.5 mg/kg as a rescue treatment for bladder discomfort reduction [6], and postoperative tramadol requirement will be recorded and compared.
2. Postoperative pain will be evaluated by the patients themselves using a single 11-point Numeric Rating Scale (NRS), which ranges from 0 to 10, where 0 indicates “no pain” and 10 represents “worst imaginable pain” [17]. If the patient complains about pain with an NRS score greater than 4, intravenous sufentanil 5 μg will be administered as a rescue analgesic treatment, and postoperative sufentanil requirement will be recorded and compared.
3. Postoperative side effects will be recorded and analyzed. These include postoperative nausea/vomiting, sedation, dizziness, and dry mouth. Sedation will be assessed using the Ramsay Sedation Scale (1 = anxious, agitated, or restless; 2 = cooperative, oriented, and tranquil; 3 = responds to command, asleep; 4 = brisk response to light glabellar tap or loud noise; 5 = a sluggish response to light glabellar tap or loud noise; and 6 = no response) [18]. Patients with a Ramsay Sedation Scale score greater than 4 will be considered sedated.
4. Acceptance of the indwelling urinary catheter will be evaluated by using an NRS score ranging from 0 (totally not acceptable) to 10 (totally acceptable) after extraction of the catheter.
5. The incidence of complications related to PNB, including hemorrhage and/or hematoma, infection of injection site, and accidental systemic administration of ropivacaine, which will be recorded and evaluated for overall safety of this technique, will be recorded.

### Statistical analysis

An intention-to-treat analysis will be performed to compare all primary and secondary outcomes with the use of SPSS version 18.0 software (SPSS, Inc., Chicago, IL, USA). Demographic data will be compared using Student’s *t* test. The incidence of CRBD and postoperative side effects will be analyzed by chi-square test. The severity of CRBD will be analyzed using Fisher’s exact test. NRS scores for postoperative pain and acceptance of the indwelling urinary catheter will be analyzed using the Mann-Whitney *U* test. Postoperative tramadol and sufentanil requirements will be analyzed using the *Z* test. *p* < 0.05 will be considered to be statistically significant.

## Discussion

PNB, a local anesthetic technique, has been applied widely in patients undergoing perineal and vaginal surgery [13–15], for relief of pudendal neuralgia [16], and for treatment of detrusor-sphincter dyssynergia and external urethral sphincter hypertonicity in patients with spinal cord injury [19, 20]. In addition, a combination of pudendal and periprostatic nerve block has been reported to improve pain reduction during transperineal prostate biopsy compared with periprostatic anesthesia only [21]. On the basis of findings reported in the literature and the neural anatomy that sensation of the bladder triangle and urethra provided by the pudendal nerve derived from sacral nerves S2–S4, we hypothesize that PNB could reduce the incidence of CRBD as well as side effects due to less nerve-related side effects associated with nerve block than intravenous systemic administration of antimuscarinic agents.

As a weak opioid with a potent antimuscarinic effect, tramadol is commonly used for postoperative pain relief and chronic pain management [22, 23], and it is reported to halve the incidence of postoperative CRBD [6] and is administered to patients with severe CRBD as a rescue treatment [9]. Therefore, we chose intravenous tramadol administration as the control and will test the superiority of PNB with 0.33 % ropivacaine to tramadol 1.5 mg/kg in CRBD prevention. On the basis of a previous study [9], intravenous tramadol 1.5 mg/kg will be given to the patients with moderate to severe CRBD as a rescue treatment to relieve their bladder discomfort.

During the execution of the PNB trial, all eligible patients will receive the allocated intervention while under general anesthesia, and the research nurses responsible for follow-up and the statistician will not be aware of to which group the patient or data belongs, so the patients, the research nurses, and the statistician will be blind to the group allocation. A 16-French Foley catheter will be inserted into the patients in the present study to test the effects of CRBD prevention because this diameter of the Foley catheter is superior to the 18-French diameter and has been reported to be an independent predictor responsible for moderate or severe CRBD [1]. Sterilized liquid paraffin will be applied for catheterization for prevention against urethral mucosal injury, but local anesthetics will not be spread onto the surface of catheter, to minimize other confounding factors related to CRBD. Furthermore, although we will test the superiority of PNB with ropivacaine to intravenous tramadol in CRBD prevention, we do not aim to deny the beneficial effects of antimuscarinic agents to relieve CRBD. Instead, we will use tramadol as a rescue treatment to reduce bladder discomfort in the PACU.

There are several limitations of this trial. First, with regard to the study design, because of different routes of administration (nerve block vs. intravenous), the investigator and responsible physicians will not be blind to group allocation. Second, PNB will be performed with the patient in lithotomy position in current clinical practice, so we will choose patients undergoing prostate surgery placed in the lithotomy position. Therefore, a limitation exists in the application of PNB to patients undergoing other operations in the supine, prone, or other position, and other routes of PNB should be explored in the next step. Third, for patients undergoing medical procedures requiring urinary catheterization without any surgical intervention, the risks and benefits of PNB should be balanced, and this aspect will not be discussed in this study. In addition, CRBD is a very subjective feeling, and it can be defined only semiquantitatively on the basis of the patient’s own complaint and the physician’s judgment. That is why the incidence of CRBD varies in different articles [1, 3], and we will try our best to get an accurate measurement of CRBD by careful follow-up.

In summary, this trial is designed to find an optimal clinical practice for CRBD reduction by performing a superiority test for the PNB with 0.33 % ropivacaine compared with intravenous tramadol 1.5 mg/kg.

## Trial status

The study is not yet recruiting as of the date of publication.

## Additional file

Additional file 1: Standard Protocol Items: Recommendations for Interventional Trials (SPIRIT) 2013 checklist: recommended items to address in a clinical trial protocol and related documents. (DOC 117 kb)

## Abbreviations

CONSORT: Consolidated Standards of Reporting Trials
CRBD: Catheter-related bladder discomfort
PNB: Pudendal nerve block
NRS: Numeric Rating Scale
PACU: Postanesthesia care unit
SPIRIT: Standard Protocol Items: Recommendations for Interventional Trials
